# Authors’ Response to Peer Reviews of “Automating Individualized Notification of Drug Recalls to Patients: Complex Challenges and Qualitative Evaluation”

**DOI:** 10.2196/82609

**Published:** 2026-01-13

**Authors:** Meghana Gadgil, Rose Pavlakos, Simona Carini, Brian Turner, Ileana Elder, William Hess, Lisa Houle, Lavonia Huff, Elaine Johanson, Carole Ramos-Izquierdo, Daphne Liang, Pamela Ogonowski, Joshua Phipps, Tyler Peryea, Ida Sim

**Affiliations:** 1 Division of General Internal Medicine, University of California, San Francisco, Box 0320, San Francisco, CA, 94143, United States, 1-415-353-7922; 2 Division of Cardiology, University of California, San Francisco, San Francisco, CA, United States; 3 Clinical and Translational Science Institute, University of California, San Francisco, San Francisco, CA, United States; 4 Food and Drug Administration, Silver Spring, MD, United States; 5 Tuvli, LLC, Herndon, VA, United States; 6 Conceptant, Inc, Falls Church, VA, United States

**Keywords:** notification system, drug recalls, patient safety, medication, electronic health records, prescriptions, decision support

*This is the authors’ response to peer review reports for “Automating Individualized Notification of Drug Recalls to Patients: Complex Challenges and Qualitative Evaluation.”*

## Round 1 Review

### Reviewer E [1]

#### General Comments

*This paper [* *2* *] describes a qualitative study that aims to leverage the US Food and Drug Administration’s (FDA’s) Healthy Citizen prototype platform, which provides information about recalls, to automatically notify patients of relevant recalls.*

#### Specific Comments

##### Major Comments

*1. Because of the setup of this document, it is challenging to be able to add comments or do any editing. Not sure what happened, but it treated every line as a single object when opened in Microsoft Word. Please check your formatting.*

**Response:** We’re sorry reviewing the document was difficult; we uploaded the document following the instructions provided. We hope the problem will not present itself again in the revised document uploaded after the review.

*2. On page 2, within the abstract, under Background, there is an error in the formatting. There should be a section that begins with Aim. Instead, that section is folded into the Background section and needs to be corrected.*

**Response:** Thank you for the comment. This has been corrected in the revised manuscript.

*3. On page 8, with the MyChart message, I can see why patients felt too much wording was in this layout. Surprisingly, the Patient Advisory Council agreed to this layout and the wordiness. The focus must be on the patient’s needs, not what the FDA requires. We all have seen the Prescribers' Digital Reference, and we know that the information is too dense and too small. This is similar to that in terms of format. Enlarge the font, eliminate extraneous information, and only include information that is important to the patient and in simple English. This should be pretty feasible in the formatting of the Healthy Citizen and/or the MyChart message.*

**Response:** Thank you for the comment. We agree that clear and concise information is key in any communication with the patient. The design balanced FDA requirements, the information available on the Healthy Citizens platform, and the need to be accurate. As for the font size, please consider that the figure is an artifact, as the original screenshot needed to be shrunk to fit onto the manuscript page.

*4. You identified problems and that patients would feel obligated to contact their provider regarding the recall. Instead of exploring how to address this so that patients wouldn’t do that, thereby increasing the significant workload on the provider’s health care team, you simply gave up. I think you could have done much more with this than say, “oh, it can’t be done.” How could you word the MyChart to direct the patients only to the pharmacy that dispenses their medication instead of the primary care provider? If you didn’t ask that question, you should have. This is not the time to give up. It’s time to inquire more to find the right answers so that this could move forward and better serve both the patients and their providers.*

**Response:** Thank you for the comment. The MyChart message refers the patient only to the dispensing pharmacy and never mentions the prescribing physician or the clinic. However, during the qualitative evaluation, it became clear that the patients still wanted to discuss the recall with their clinicians. We felt that stronger wording, something along the lines of “Please do not contact your physician or the clinic on this matter,” would have been detrimental and turned patients off.

*5. It is certainly possible, given the technical requirements to create this capability, that you ran out of time and money. However, you can still benefit your team and others by focusing on the lessons learned and how you would go forward with another study.*

**Response:** Thank you for your comment. As detailed in the paper, recall alerts sent to patients are not precise, but contacting the right patient for the appropriate recall is of paramount importance to avoid unnecessary anxiety and, worse, treatment discontinuation. False positives and the fact that patients expect their prescriber to be aware of, and involved in, responding to a drug recall, while prescribers don’t have easy access to the relevant information, create an obstacle that another study would ultimately encounter and currently not be able to solve.

*6. One of the things that you did not do is a first round of qualitative testing and using that feedback to make changes and do a second round. Per Nielsen [* 3 *], you only need about 5 test subjects per round to get the desired, usable results. What was preventing you from doing that? Put that in the manuscript as a limitation in your Discussion.*

**Response:** Thank you for your comment. The study was planned based, among other things, on a project timeline. See the answer to comment 5 above.

*7. Also, on page 11, in the last paragraph of the page under Discussion, there is a comment regarding patients expecting their providers to know when a recall has occurred; I think we all know this is an unreasonable expectation. Part of the communication with the MyChart message is to inform the patients not to call their provider but to call the pharmacy that dispenses their medication, which should be right on the bottle. Again, one component of the MyChart portal messaging system, as well as any other portal messaging system, is to keep patients informed and educate them. That should be a focus of this project, just as much as the technical components.*

**Response:** Thank you for your comment. As mentioned in our answer to comment 4 above, we feel that a stronger wording, something along the lines of “Please do not contact your physician or the clinic on this matter,” would have been detrimental and turned patients off.

*8. On page 12, in the last full paragraph on the page, you make a statement regarding the project that a strong case can be made for requiring each pill bottle to include the lot number (maybe) and National Drug Code (NDC) of the pills. Since the FDA was a component of this project, that should probably have been something you recommended for the FDA to require and not leave to the state boards of pharmacy, as then you would get a patchwork of regulations. This would require the FDA to say that lot numbers and NDCs are required on the bottles of all medications with an appropriate implementation period to allow for appropriate software and hardware adjustments. That is just as valuable a recommendation out of the study as any other.*

**Response:** Thank you for your comment. The FDA does not have the legal authority to regulate the practice of pharmacy in any state, and therefore the FDA cannot require that the lot number and NDC (or anything else, including the name of the drug) be placed on each prescription that a pharmacist dispenses to a patient. We clarified this in the Discussion section of the revised manuscript.

### Reviewer F [4]

#### General Comments

*This manuscript [* 2 *] describes interesting and novel work with far-reaching patient safety implications. The authors developed an automated system in the electronic health record (EHR) of an academic medical center that scans for drug recalls, matches up NDCs of recalled medication on a patient’s medical list, and sends notifications through the EHR portal to the patient, providing them with more information on the recall. The authors then conducted a qualitative analysis of 9 patients’ perceptions of a fictious recall notice. Despite successful development of the automated system, many limitations prevented the widescale adoption of this system in 2 clinics associated with the large academic medical center. The outcome of the work—a decision was made not to deploy the new software for drug recalls—was surprising, and it is important that “failed” implementation work also be published. That said, key weaknesses of the manuscript are the lack of important details, need for better organization of the content, and the need for much stronger scientific and technical writing to accurately interpret the methods, results, and implications. These weaknesses also made it much more difficult to read and evaluate the manuscript. Despite the importance of the topic, the small sample size of patients also limits the work’s impact.*

#### Specific Comments

##### Title

*1. It would be helpful if the title were a bit more specific about the technology, study methods (qualitative), and notification recipients (patients, providers, etc).*

**Response:** Thank you for the suggestion. We edited the title to Automating Individualized Notification of Drug Recalls to Patients: Complex Challenges and Qualitative Evaluation.”

##### Abstract

*1. The Background section appears to be contradictory. Sentence 2 says the FDA has ways to notify health care professionals (HCPs) and patients, but then the following sentences seem to say the opposite.*

**Response:** Thank you for your comment. The website referenced in the paper, “Recalls, Market Withdrawals, & Safety Alerts” [5], provides information to the public about recalls, but it does not notify HCPs about individual patients in their care who may be affected.

*2. A few more details here on the type of platform would be helpful...software app? Web-based platform, etc? And what are the intended user types? (HCPs and patients? Or just patients?)*

**Response:** Thank you for your comment. We added some details to the abstract.

*3. The choice of methods doesn’t seem to follow the Background section. Why was it necessary to include the clinics, rather than just work directly with the patients? Or, why was the focus on clinics, rather than pharmacies? (These comments apply to the main Introduction and Methods sections, as well.)*

**Response:** Thank you for your question. As the study was implemented at an academic institution, we followed the institution’s rules for engaging with patients.

*4. I expected the “program description” to appear in the Methods section, not the Results.*

**Response:** Thank you for your comment. The section was moved as suggested.

##### Introduction

*1. The second and third sentences of the first paragraph of the Introduction: any studies or references to back up this claim?*

**Response:** Thank you for your question. The paper’s authors, who are HCPs, have extensive experience managing drug recalls in their daily practice. Published studies have focused on analyzing and classifying the recalls themselves (eg, the reason for the recall, the class).

*2. No information is included on if/what literature explores this or similar topics.*

**Response:** Please see the response to comment 1 under Introduction above.

*3. I would recommend adding more information on the process pharmacies currently have in place for notifying patients of recalls. Also add any literature that exists showing how often patients then contact their providers or add quantitative data to highlight this extra burden on providers to emphasize the problem.*

**Response:** Thank you for your comment. The process pharmacies follow is explained in the Drug Recall Process section. We found no literature on the topic.

*4. I expected the funding information in the last sentence of the first paragraph to be included in a funding statement or the acknowledgments (rather than the Introduction) and the rest of that statement to be described in the Methods.*

**Response:** Thank you for your comment. We moved the paragraph as suggested.

##### Setting

*1. I expected this to appear under a larger Methods section.*

**Response:** We added a Methods section.

*2. What was the goal sample size and rationale for the sample size? There is missing demographic information on the participating patients.*

**Response:** A convenience sample was used based on outreach to patients and their responses. Given the nature of the study, we feel we provided sufficient information on the patients interviewed.

*3. So the Fast Healthcare Interoperability Resources (FHIR) portion notified HCPs? The intended recipients are not specified for that part of the program.*

**Response:** The notification was meant for patients only.

*4. “EHR build” was unexpected as a reader. Is that a third part? How does it fit into the first 2 parts?*

**Response:** We added a clarification.

*5. The screenshots and figures are useful.*

**Response:** Thank you for your comment. We are glad the figures are useful.

*6. Even for a convenience sample, more details are needed on recruitment. How did you choose which patients to email? How many were emailed for recruitment? Were patients emailed and recruited sequentially, for example? Were there any exclusion or inclusion criteria for patients? Did any patients decline to participate? Why? What was the distribution of patients recruited from primary care versus cardiology?*

**Response:** Recruitment techniques were not a subject of this study. Patient inclusion was based on active use of the MyChart portal.

*7. More specific details are warranted for the methods used for qualitative analysis, such as whether an inductive versus a deductive design was used. Was a consensus approach used, or some other approach? See also the writing guidelines for qualitative studies (eg, the Consolidated Criteria for Reporting Qualitative Research [COREQ], Standards for Reporting Qualitative Research [SRQR]). Explain also the “additional verification” process during analysis. References should be cited for the qualitative methods used in this work.*

**Response:** We added the interview script as an appendix. Analysis of the responses was repeated and the results compared.

*8. Did any of the patient sample have prior experience with MyChart, and if so, what was the average number of years of MyChart experience?*

**Response:** Active use of MyChart was an inclusion criterion.

*9. These statements from the text appear to be contradictory, and the meaning of the first statement especially is unclear, and seems like an opinion: “[Patients expressed that the] widget should not ask patients to discuss the information with their healthcare provider.” “Patients wanted to discuss the recall with their clinicians to ‘close the loop.’”*

**Response:** Figure 2 shows the information provided by the FDA’s Healthy Citizen platform, which complies with the FDA’s requirements and is not customizable by the system using it.

*10. The conclusion not to deploy the system seems dramatic based on the findings and makes me wonder if any other creative solutions were considered to address the concern of potential increased clinic burden. Also, how was it determined that the clinic burden outweighed safety risks to the patient? Maybe the system should only be used for certain types of recalls, for example. Or maybe the system could be integrated more with the pharmacy, rather than the prescriber’s clinic, or the letter could read differently (advising against contacting the clinic unless the patient was unable to resolve the issue with the pharmacy). Or the letter could explain that only the pharmacy, not the clinic, would have a record of the patient’s specific manufacturer and whether the recall applied to them.*

**Response:** The MyChart message explains that the pharmacy has more information about the drug given to the patient; the message cannot state for certain that the pharmacy can match the recall precisely to the patient.

*11. It would be helpful to see the full interview guide and patient scenario details in a supplementary appendix to aid interpretation of the methods and results.*

**Response:** We added the interview script as a multimedia appendix.

##### Discussion

*1. The Discussion does not mention limitations of the study design and methods.*

**Response:** Our project was technically successful. The lack of availability of the data needed to accurately target patients—particularly the lot number of the drug dispensed—makes false positive notifications unavoidable, independent of the study design.

*3. Is anything stamped on the medication (eg, pill) itself to indicate the manufacturer? Or is that also inconsistent across medications?*

**Response:** As described in the Discussion section, what data pertaining to the drug appears where is not consistent. (And while the pharmacy records the NDC of filled prescriptions, pills from different lot numbers can be dispensed together.)

*5. In the last paragraph of the Discussion, there is no citation for the number of state boards of pharmacy that require the lot number to appear on the label.*

**Response:** While we understand the interest in learning which state boards of pharmacy require the lot number to appear on the label, considering that the number is one-tenth of the states, the takeaway is that the problem described applies to the vast majority of the states.

*6. I expected the Discussion to close with a Conclusions paragraph outlining key lessons learned and any generalizable findings.*

**Response:** In the Conclusions we reiterate the need for consistent availability of the data needed to accurately address patients affected by a drug recall.

## Round 2 Review

### Reviewer E

#### General Comments

*This paper describes a small qualitative study that aims to leverage the FDA’s Healthy Citizen prototype platform, which provides information about recalls, to notify patients of relevant recalls automatically. The project team deemed the goal unattainable and provided limited lessons learned and recommendations for potential advocacy/future solutions.*

#### Specific Comments

##### Major Comments

*1. On page 11, in the section/paragraph beginning with “Major thematic findings included...”: these are some of the lessons learned that I mentioned in my feedback.*

**Response:** Thank you for your suggestion. We added content to the Conclusions paragraph summarizing lessons learned and outlining the generalizability of some of them.

*2. On page 12, in the paragraph beginning with “The project team concluded that...”: The “project team” felt this. Did the Patient Advisory Council and the test subjects share the same feeling?*

**Response:** Thank you for your question. We did not go back to the Patient Advisory Council or to the test subjects after the conclusion of the project. While their support for expanding the pilot would have been encouraging, their support could not solve the challenges encountered during the project implementation. An expansion would have required institutional support. While the project implementation provided important lessons, it did not provide a solid enough business case to justify expanding the pilot. We added this to the Program Evaluation section.

*3. On page 13, in the second paragraph on the page, in the sentence beginning with “Note that the FDA does not...”: this would clearly be a lesson learned and could be advocated for via Congress and the Department of Health and Human Services.*

**Response:** Thank you for your comment. We added the following content to the Conclusions paragraph to address it: “While a change at the federal level would be ideal, advocating individual State Boards of Pharmacy to require the NDC and lot number to appear on the dispensed medication label may provide interim needed progress allowing development and deployment of solutions supporting patients’ needs.”

*4. On page 13, in the the second paragraph, the next sentence, beginning with “The manufacturer and lot number of dispensed medications...”: agreed. See previous comment.*

**Response:** Thank you for your comment. We added content to the Conclusions paragraph to address it. See our response to comment 3 above.

### Reviewer F

#### General Comments

*The authors addressed a few of my review comments and made some text changes, but unfortunately, most of my comments—about 15 of them—remain inadequately addressed. For the comments listed again below, the authors did not appear to change anything in the manuscript to address the comment. In many cases, even the authors’ reply to the reviewers did not answer the question. Also, the authors describe adding the interview guide as an appendix, but I could not find this file on the reviewer website.*

**Response:** Regarding the interview guide: the file was uploaded on April 12 on the authors’ submission website, ahead of the resubmission and recirculation of the manuscript. Right-clicking on the file name identifies the URL [6].

#### Specific Comments

##### Abstract

*1. The Background section appears to be contradictory. Sentence 2 says the FDA has ways to notify health care professionals (HCPs) and patients, but then the following sentences seem to say the opposite.*

**Response**: The FDA has public-facing resources, including the Recalls, Market Withdrawals, & Safety Alerts website [5], which can be consulted by anyone. However, as mentioned in the Abstract, prescribers are not notified individually and specifically about which of their patients are affected by a recall. We added some words to clarify the distinction between general and specific and deleted the last sentence.

*3. The choice of methods doesn’t seem to follow the Background section. Why was it necessary to include the clinics, rather than just work directly with the patients? Or, why was the focus on clinics, rather than pharmacies? (These comments apply to the main Introduction and Methods sections, as well.)*

**Response:** The project’s premise was that patients seek answers to recall-related questions from their HCPs. Therefore, we wished to answer the question at the levels of primary care and a cardiology clinic. We worked with the project principal investigators’ clinics and patients and did so following the applicable requirements.

##### Introduction

*2. No information is included on if/what literature explores this or similar topics.*

**Response:** We added 4 references, 3 of them to recently published papers focused on the analysis of recall-related data (see the response to question 2 under Discussion below for summary details]

##### Setting

*2. What was the goal sample size and rationale for the sample size? There is missing demographic information on the participating patients.*

**Response:** As previously noted, the convenience sample was based on outreach to patients and their responses. Given the exploratory nature of the study, we feel we provided sufficient information on the patients interviewed. Power calculation and balancing the sample for certain variables were not relevant.

*3. So the FHIR portion notified HCPs? The intended recipients are not specified for that part of the program.*

**Response:** Thank you for your question. No, the HCPs did not receive any notification. The Healthy Citizens (SMART-on-FHIR) widget was launched from the MyChart message sent to the patient. We added a sentence between Figures 1 and 2 to clarify.

*6. Even for a convenience sample, more details are needed on recruitment. How did you choose which patients to email? How many were emailed for recruitment? Were patients emailed and recruited sequentially, for example? Were there any exclusion or inclusion criteria for patients? Did any patients decline to participate? Why? What was the distribution of patients recruited from primary care versus cardiology?*

**Response:** Thank you for your questions. We added some details to the manuscript in response. Established patients at the Department of General Internal Medicine (primary care) clinic who were members of the Patient Advisory Council, used MyChart, and were prescribed at least one medication received a recruitment letter. Patients at the cardiology clinic who were scheduled to see the pharmacist during a random week, who actively used MyChart (or their family members who used MyChart on their behalf), and who used at least one prescription medication were deemed eligible for the study and sent a recruitment letter. Interested patients contacted the study team to participate. Nine patients were interviewed.

*7. More specific details are warranted for the methods used for qualitative analysis, such as whether an inductive versus a deductive design was used. Was a consensus approach used, or some other approach? See also the writing guidelines for qualitative studies (eg, the COREQ, SRQR). Explain also the “additional verification” process during analysis. References should be cited for the qualitative methods used in this work.*

**Response:** Thank you for your question. The objective of the interviews was to obtain qualitative feedback from patients and identify the feedback’s main themes using a consensus approach (a reference has been added to the manuscript). As detailed in the manuscript, the recordings of the interviews were transcribed and separately analyzed by 2 investigators to identify common themes, then 2 other team members verified the initial analysis. These themes are described in the manuscript in the paragraph starting with “Major thematic findings included the following...”

*8. Did any of the patient sample have prior experience with MyChart, and if so, what was the average number of years of MyChart experience?*

**Response:** The 9 patients interviewed were all MyChart users. We clarified in the manuscript that MyChart use was an inclusion criterion. We did not consider the number of years of MyChart experience as a relevant data point.

*9. These statements from the text appear to be contradictory, and the meaning of the first statement especially is unclear, and seems like an opinion: “[Patients expressed that the] widget should not ask patients to discuss the information with their healthcare provider.” “Patients wanted to discuss the recall with their clinicians to ‘close the loop.’”*

**Response:** The suggestion to discuss the recall information with the health practitioner was displayed on the FDA Health Citizen widget and could not be modified. We clarified this in the manuscript. The interviews confirmed that the statement led to confusion. The MyChart message recommended calling the pharmacy, as it would be the entity with more information to help the patient verify whether the recall applied to them (Figure 1).

*10. The conclusion not to deploy the system seems dramatic based on the findings and makes me wonder if any other creative solutions were considered to address the concern of potential increased clinic burden. Also, how was it determined that the clinic burden outweighed safety risks to the patient? Maybe the system should only be used for certain types of recalls, for example. Or maybe the system could be integrated more with the pharmacy, rather than the prescriber’s clinic, or the letter could read differently (advising against contacting the clinic unless the patient was unable to resolve the issue with the pharmacy). Or the letter could explain that only the pharmacy, not the clinic, would have a record of the patient’s specific manufacturer and whether the recall applied to them.*

**Response:** The MyChart message recommended calling the pharmacy as it is the entity with more information to help the patient verify whether the recall applied to them (Figure 1). Patients contacting the clinic received the same instructions. Most pharmacies have protocols in place to handle recalls, which may include outreach to customers. Integration with pharmacies was out of scope for this project and would have been a substantial undertaking: just the 2 clinics involved in the project serve over 37,000 patients, who fill their prescriptions in different pharmacies, from large chains to small local pharmacies to online ones. In the manuscript, we mention integrating with Surescript via claims data. However, such integration would not cover all the institution’s patients, and Surescript records do not include dispensed lot numbers, so the problem of false positive notification would still exist. Should funding become available, we do not rule out exploring alternative solutions in the future. In response to a comment from the other reviewer, we added in the Program Evaluation section that while the project implementation provided important lessons, it did not provide a solid enough business case to justify expanding the pilot, which would have required institutional support.

##### Discussion

*1. The Discussion does not mention limitations of the study design and methods.*

**Response:** The project did not move forward for reasons that go beyond the qualitative evaluation we performed (see also our response to comment 10 under Setting, above).

*2. I expected at least some comparison to other, related literature.*

**Response:** We added references to recently published papers:

An analysis of FDA drug recall data (2012-2023) showing that drug recalls are frequent [7]. The paper talks about the causes of drug recalls and suggests improvements to the relevant FDA database, but it doesn’t discuss the impact of recalls on clinical care.

A study of drug recalls in the Netherlands, which also identifies the issue that pharmacists do not always know which batch was dispensed to a patient [8].

An analysis of the clinical impact of the 2018 recalls of several angiotensin II receptor blockers and the impact in terms of medication gap and clinical outcomes [9].

These are recently published supporting articles that analyze existing data. None includes a program such as ours.

*3. Is anything stamped on the medication (eg, pill) itself to indicate the manufacturer? Or is that also inconsistent across medications?*

**Response:** What is printed on an individual solid oral-dosage-form product (eg, tablet or capsule) depends on the manufacturer complying with 21CFR206.10(a) in the Code of Federal Regulations [10]. In the United States, most solid oral-dosage-form drug products are required to have an imprint code (eg, logo, letters, numbers, or a combination). As detailed in the manuscript, at the federal level, the FDA does not have the legal authority to regulate the practice of pharmacy in any state and cannot require that specific information be placed on each prescription label that a pharmacist dispenses to a patient. Individual states (via their state boards of pharmacy) regulate what appears on the pill bottle label and on the leaflet provided to the patient alongside the medication.

*4. A table of key recommendations could strengthen the paper.*

**Response:** Thank you for your suggestion. We have added a list of lessons learned and a recommendation at the end.

*5. In the last paragraph of the Discussion, there is no citation for the number of state boards of pharmacy that require the lot number to appear on the label.*

**Response:** We added the requested details to the statement pertaining to lot number requirements and added the relevant supporting references. No peer-reviewed synthesis exists on this point, so we relied on primary legal sources. We also amended the original statement pertaining to the NDCs to clarify the rules and the issuing body: “As of August 2025, our review of state regulations identified the following jurisdictions with explicit requirements. Four State Boards of Pharmacy (Colorado, Delaware Oklahoma, Wyoming) plus the U.S. territory of Puerto Rico require the lot number to appear on the dispensed medication label [12-16]. The Pennsylvania State Board of Medicine requires the NDC to appear on the dispensed medication label if the prescriber specifies that the drug name not appear on the label [17]. The State Boards of Pharmacy of New Hampshire and Ohio, allow the use of NDC as abbreviation for the manufacturer / distributor name [18-19].”

## Abbreviations

COREQ: Consolidated Criteria for Reporting Qualitative Research
EHR: electronic health record
FDA: Food and Drug Administration
FHIR: Fast Healthcare Interoperability Resources
HCP: health care professional
NDC: National Drug Code
SRQR: Standards for Reporting Qualitative Research
